# Exploring Cambodian adolescents' perceptions on sex: a qualitative investigation

**DOI:** 10.3389/frph.2024.1275941

**Published:** 2024-05-16

**Authors:** Youngran Yang, Jiwoo Kim, Gloria Park, Roshna Thapa

**Affiliations:** ^1^ School of Nursing, Research Institute of Nursing Science, Sustainable Development Center, Jeonbuk National University, Jeonju, Republic of Korea; ^2^ Seoul Samsung Medical Center, Seoul, Republic of Korea; ^3^ St. David’s School of Nursing, Texas State University, Round Rock, TX, United States; ^4^ Jersey City Medical Center, Jersey City, NJ, United States

**Keywords:** adolescents, cambodia, HIV, sexual health, STIs

## Abstract

**Introduction:**

Involvement in sexual activities increases during adolescence in many countries, including Cambodia. The objective of this study is to explore the perspectives and interpretations of sex held by Cambodian adolescents within the context of their social norms and culture.

**Methods:**

A qualitative research design was used to conduct in-depth interviews with a purposive sample of 91 Cambodian adolescents aged between 15 and 19 years. Participants were recruited from rural areas, and data was collected through face-to-face interviews using semi-structured interview guides. Thematic analysis was used to analyze the data.

**Results:**

Four themes as perspectives of sex were identified: (1) Desire: Releasing sexual desire and stress; (2) relationship: an emotional connection and demonstration of love; (3) roles: responsibilities within a woman's marital duties; and (4) values: the value of virginity and future engagement. Cambodian adolescents' perspectives and interpretations of sex were deeply influenced by their social norms and cultural values. Men typically perceived sex through the lens of instinct and pleasure, while women often emphasized a deep sense of familial duty and held premarital sex to be morally unacceptable.

**Discussion:**

The findings suggest that interventions aimed at improving the sexual health of Cambodian adolescents should be designed with an understanding of the social norms and cultural values that shape their perspectives and interpretations of sex. Such interventions should focus on promoting safe sex practices and providing accurate and comprehensive sexual education.

## 1 Introduction

Adolescence is the period between 10 and 19 years of age that marks the transition from childhood to adulthood (1). Adolescents experience rapid changes not only in their physical growth but also in cognitive, emotional, and psychosocial development while pursuing sex and intimate relationships (1). Adolescence is considered a healthy period of life; however, negative sexual and reproductive health (SRH) outcomes can threaten the well-being of adolescents, especially in low- and middle-income countries (2). Young people typically engage in sexual activities as they reach adolescence, with limited knowledge about SRH, leading to the highest rates of sexually transmitted infections (STIs) and HIV infections (2).

The global adolescent population has increased, with the majority of adolescents living in Southeast Asia (3). Cambodia has the largest adolescent and young adult population in Southeast Asia, with two-thirds of its 14.7 million people under the age of 30 (4). However, Cambodia lags behind its neighboring countries in implementing effective strategies to improve SRH. Cambodian youth face numerous obstacles to sustainable SRH, including lack of SRH literacy and limited access to modern contraceptives (4). Only 6.7% of Cambodian youth reported having visited a local health center, hospital, or clinic to seek reproductive health care (3). Furthermore, the rate of condom use among young Cambodian men decreased from 26% in 2010 to 18% in 2014 (5). This means that challenges, such as unexpected pregnancies and STIs, are on the rise.

However, essential health services are expensive, particularly in rural areas, due to user fees and transportation, as well as food and accommodation costs. Geographical factors, including the time required to travel to facilities and transportation availability, are barriers to health care access (6). Therefore, eliminating financial and geographical barriers is critical to increasing health care utilization. In addition, sex education must be taught among adolescents, particularly on how to practice safe sex. Since mid-1997, the Reproductive Health Association of Cambodia has used peer educators, group discussions, one-on-one discussions, local theaters or quiz shows, various educational materials, and youth centers to convey reproductive health messages and information to young people. In 2007, the project distributed condoms and offered STI services to youth under the age of 25 (7). However, condom use declined over the following four years (5). This implies that the program must be revised the program to boost its effectivity.

Sex is often linked to the concept of marriage, with some regarding it as conditional on marriage, especially in rural areas. Young, unmarried individuals in urban Cambodia are 60% more likely to engage in pre-marital sexual intercourse compared with those who live in rural areas (5). Our study also supports this finding, stating that many of the respondents from rural Cambodia oppose premarital sex. After marriage, the purposive act of having a child has emerged as another primary reason for sex. Most rural households depend on agriculture and related subsectors to survive. Since launching its official rice export policy in 2010, Cambodia has emerged as a major player in the international rice commodity trade (8). Therefore, agricultural human resources are necessary for survival, and children are a form of accessible and inexpensive labor.

Cambodia's traditional wedding culture welcomes forced marriages and teenage pregnancies. For many Cambodians, marriage enhances their social and economic status (9). In addition to robbing a girl of her childhood, education, and future independence, child marriage also exposes her to the risk of fatal health complications associated with early childbearing. Additionally, forced child marriage exposes girls to repeated sexual and physical violence, which can have devastating effects on their mental and physical health and undermine gender equality (10). This practice deprives Cambodian women of sexual agency after marriage.

Most of the social norms underpinning this marriage system are patriarchal. For young girls, early marriage signifies an early transition to adulthood, socially imposed sexual norms, mandatory obedience, filial piety, and lack of economic freedom (11). In Cambodia, women are constantly exposed to sexual violence as a result of their subordinate status in a patriarchal society. This system renders women vulnerable to exploitation at the hands of their husbands, fathers, neighbors, authorities, and other male figures (12). Furthermore, social and cultural practices prevent women from exercising their rights to self-determination. These social norms are linked to Cambodia's early and forced marriage systems (5), as well as the Khmer cultural principle known as *Chbab Srey* (13). Cambodian culture encourages marriage at a young age, when women are typically unable to decide for themselves. *Chbab Srey* is considered crucial in Khmer culture and is taught in schools and Khmer literature. It codifies women's status at home and conveys the idea that married women should be respectful and submissive toward their husbands (13). Failure to comply with *Chbab Srey* results in social sanctions and exclusions (14). Moreover, most women are dependent (financially or otherwise) on their husbands, especially if they have children. In this social system, women have no option but to obey men (12).

Essentially, having children means preparing for old age. In Cambodia, filial piety means that most residents believe their children should devote themselves to their parents' welfare.

Many Asian cultures view sexuality as taboo and forbid sexual activities outside of marriage. However, increased access to media access has strengthened permissive attitudes toward dating and premarital sex among adolescents (2). In Cambodia, men enjoy more freedom than women. Women in Cambodia often repress their potential, whereas men enjoy the innate privileges afforded by their gender (5). A famous Cambodian Khmer proverb, “fruits should not ripen before they change color,” advises young women to maintain their virginity until marriage (15). In Cambodia, a woman's virginity is considered a sacred virtue reserved for their future spouses. Furthermore, a girl who loses virginity is a disgrace to her family, regardless of whether it was caused by sexual abuse, and will remain a loss of “virtue” (9). Cambodian society also disapproves of children born outside of wedlock, compelling young pregnant girls to marry (12). The 1975–1979 Cambodian Civil War disrupted social and family norms and precipitated rapid lifestyle changes. This period saw a decline in traditional monogamy and increased sexual promiscuity, often resulting from increased access to sex workers, who are individuals receiving monetary compensation in exchange for consensual sexual services (16). Almost half of the participants (45.0%) had their first sexual experience with a sex worker, and over half (58.3%) had engaged in sexual intercourse with multiple sex partners, including their wives (16). In Phnom Penh, Cambodia, the prevalence of HIV among sex workers ranged from 9.2% to 23% (17). Alarming statistics revealed that over 40% of new HIV infections are identified among adolescents (18). Furthermore, most Cambodian men engage in unprotected sex, such as not wearing a condom, with their wives, despite being uncertain about their HIV status (19). Some reasons for not practicing safe sex were poor sexual sensitivity, lack of prophylactic knowledge, and the belief that condom use indicates a lack of spousal trust (19). A recent study in Cambodia revealed that 68% of young males aged 16–24 years old were sexually active, and 27% of them had sexual contact with sex workers within the previous year, placing them at a high risk of contracting HIV (20).

In a qualitative study among Cambodian adults, sex was perceived as a woman's obligation but a man's personal pleasure (21). However, the sexual behaviors and perceptions of Cambodian adolescents are gradually changing and are influenced by cultural perceptions and subjective factors. As such, the patriarchal culture practiced in Cambodia may have substantially influenced these perceptions and behaviors. The purpose and meaning of sex in adolescents may be associated with early sexual initiation and practice of unprotected sex. This may also be related to efforts to maintain virginity, all of which can help develop programs to prevent STIs and improve reproductive health. This study was conducted to explore the perceptions of Cambodian adolescents toward sex to understand the cultural influence on sexual behaviors.

## 2 Materials and methods

### 2.1 Study design, participants, and setting

A descriptive qualitative approach was used to investigate the significance of sex to Cambodian adolescents. Three rural provinces in Cambodia, Kandal, Kompong Speu, and Kampong Chhnang, were selected through convenience sampling. This study was conducted between July and August 2017 in the high schools of these provinces. To be included in this study, participants must be (a) Cambodian residents, (b) third-year high school students, (c) aged 17–19 years, and (d) unmarried. The study aims, research processes, and interview questions were approved by the Cambodian Ministry of Education, Youth, and Sports. Furthermore, the respective high school principals approved the study after they were informed of its purpose, eligibility criteria, participation process, and student recruitment. The schools provided a private room to ensure confidentiality. The interviews were digitally audio recorded with participants' permission. Ethical permission for the study was obtained from the Jeonbuk National University Institutional Human Subjects Review Committee (2017-06-014-002) and Cambodia National Ethics Committee for Health Research of the Ministry of Health.

### 2.2 Data collection

The study team included two non-Cambodian (two females) and two Cambodian (one male and one female) researchers. Third-year students were briefly introduced to the study and its purpose. The participants were informed of their right to information privacy, confidentiality, and withdrawal at any time. Only those who agreed to participate were given a date and time for the interview. Informed consent was obtained from the participants, and interviews were conducted in Khmer using an in-depth interview guide and open-ended questions, such as “Can you explain your understanding of the concept of sex?” to explore the general perception of ex among adolescents. Additional probing questions were used to elicit rich and detailed perspectives from the participant, such as (1) “Tell me more about that …”, (2) “Tell me what you meant by…”, and (3) “How did that make you feel?” Two non-Cambodian investigators completed the data collection, while the Cambodian investigator assisted as a bilingual Khmer/English interpreter throughout the process. A male interpreter assisted the male participants, whereas a female interpreter assisted the female participants. So as to reduce bias between the researchers, the primary investigator participated in the male and female interviews. The gender identity of the primary investigator (PI) was female, and she participated in interviews while maintaining a neutral stance. To mitigate potential gender-related biases in the interviews, the PI adhered to the principles of qualitative research and research ethics, ensuring transparency throughout the process. These deliberate efforts were undertaken to uphold rigorous and unbiased research practices. Given that the research team conducted the interviews while traveling together, after each daily interview, the research team gathered to share the interview progress and monitor the follow-up. Each interview lasted between 40 and 80 min, and data were collected until data saturation. As a token of appreciation, each participant was given 20,000 Cambodian riels (equivalent to US$5) in a sealed envelope upon completing the interview. A total of 48 male and 43 female students participated in the interviews.

### 2.3 Data analysis

Each participant was assigned a pseudonym to ensure their privacy and confidentiality. The digitally recorded interviews were transcribed by a native Khmer speaker and translated into English. The interviews were then analyzed using thematic qualitative analysis (22). First, data familiarity was achieved by repeatedly reading the English transcripts. The initial concepts that represented the most critical features of the data were then coded to produce themes. The codes relevant to each theme were classified into subthemes. Next, a thematic “map” was created on the basis of whether the potential themes worked with the extracted codes and entire dataset. Themes were then assigned definitions and names to refine their characteristics. Finally, a written analysis was conducted after reviewing themes related to the literature and interview questions. Thereafter, the transcripts were read and reviewed to ensure that the codes adequately represented the aims of the study in the Cambodian context. The ATlas.ti software (version 6.0) was used to mark the code.

### 2.4 Ensuring research rigor

In order to enhance the rigor of our qualitative research, we maintained a high degree of transparency and reflexivity throughout the study. Transparency was achieved by meticulously documenting and reporting our research process, allowing for the scrutiny of our methods and decision-making. This documentation included detailed notes on data collection, coding processes, and analytical decisions. Reflexivity was a critical component of our study, as we recognized the potential impact of our own perspectives and experiences on the research process. To address this, we consistently engaged in self-reflection and acknowledged our positionalities, which were used to inform our interpretation of the data. This reflexive approach helped ensure that our findings were as objective as possible.

## 3 Results

The average age of the participants was 17.9 and 18.7 years for females and males, respectively. Most participants identified as Buddhists. Their average monthly spending money was US$89 for females, whereas US$113 for males. Most participants lived with their parents (Table 1). Regarding what sex meant to them, the following themes emerged from the data analysis: (1) desire: Releasing sexual desire and stress; (2) relationship: an emotional connection and demonstration of love; (3) roles: responsibilities within a woman's marital duties; and (4) values: the value of virginity and future engagement. Table 2 displays the themes, sub-themes, and corresponding codes.

**Table 1:** Characteristics of the participants.

ID	Sex	Age	Religion	Currently Drinking	Currently Smoking	Living Arrangements	Pocket Money (USD)
1	Male	17	Buddhism	No	No	Two parents	200
2	Male	17	Buddhism	No	No	Two parents	100
3	Male	18	Buddhism	No	No	Two parents	100
4	Male	19	Buddhism	No	No	Renting House	125
5	Male	20	Christianity	No	No	Two parents	75
6	Male	18	Buddhism	No	No	Two parents	75
7	Male	18	Buddhism	No	No	Two parents	300
8	Male	17	Buddhism	No	No	Two parents	200
9	Male	19	Buddhism	No	No	Two parents	70
10	Male	20	Buddhism	Yes	No	Renting House	150
11	Male	18	Buddhism	No	No	Two parents	100
12	Male	18	Buddhism	No	No	Relatives	250
13	Male	19	Buddhism	No	No	Relatives	60
14	Male	20	Buddhism	No	No	Single parents	200
15	Male	17	Buddhism	No	No	Two parents	75
16	Male	19	Buddhism	No	No	Two parents	75
17	Male	18	Buddhism	No	No	Two parents	150
18	Male	21	Buddhism	Yes	Yes	Renting House	200
19	Male	19	Buddhism	Yes	No	Relatives	150
20	Male	20	Buddhism	Yes	No	Renting House	150
21	Male	19	Buddhism	Yes	No	Renting House	150
22	Male	20	Buddhism	No	No	Relatives	75
23	Male	20	Muslim	No	No	Two parents	100
24	Male	17	Muslim	No	No	Two parents	80
25	Male	19	Buddhism	No	No	Two parents	70
26	Male	17	Muslim	No	No	Two parents	75
27	Male	19	Muslim	No	No	Single parents	50
28	Male	17	Buddhism	No	No	Two parents	50
29	Male	19	Muslim	No	No	Single parents	150
30	Male	21	Muslim	Yes	Yes	Two parents	75
31	Male	19	Muslim	No	No	Two parents	25
32	Male	18	Muslim	No	No	Two parents	70
33	Male	20	Muslim	No	No	Two parents	100
34	Male	19	Buddhism	Yes	No	Two parents	125
35	Male	19	Buddhism	No	No	Relatives	40
36	Male	18	Buddhism	Yes	No	Two parents	50
37	Male	24	Buddhism	Yes	No	Two parents	75
38	Male	22	Buddhism	No	No	Two parents	170
39	Male	16	Buddhism	No	No	Two parents	125
40	Male	18	Buddhism	No	No	Two parents	75
41	Male	17	Buddhism	No	No	Two parents	80
42	Male	17	Buddhism	No	No	Two parents	200
43	Male	19	Buddhism	Yes	No	Two parents	50
44	Male	18	Buddhism	Yes	No	Brother	100
45	Male	19	Buddhism	Yes	No	Two parents	50
46	Male	19	Buddhism	No	No	Two parents	150
47	Male	18	Buddhism	No	No	Two parents	50
48	Male	8	Buddhism	No	No	Two parents	100
49	Female	18	Buddhism	No	No	Two parents	50
50	Female	17	Buddhism	Yes	No	Two parents	150
51	Female	18	Buddhism	No	No	Relatives	150
52	Female	18	Buddhism	No	No	Two parents	75
53	Female	18	Buddhism	No	No	Two parents	45
54	Female	18	Buddhism	No	No	Two parents	150
55	Female	18	Buddhism	No	No	Relatives	75
56	Female	18	Buddhism	No	No	Two parents	75
57	Female	19	Buddhism	No	No	Two parents	40
58	Female	19	Christianity	No	No	Catholic Sister	30
59	Female	19	Buddhism	Yes	No	Orphanage	7.5
60	Female	18	Christianity	No	No	Catholic Sister	25
61	Female	18	Buddhism	No	No	Two parents	200
62	Female	18	Buddhism	No	No	Two parents	150
63	Female	17	Buddhism	No	No	Two parents	100
64	Female	17	Buddhism	No	No	Two parents	200
65	Female	17	Buddhism	No	No	Two parents	200
66	Female	18	Muslim	No	No	Two parents	130
67	Female	18	Muslim	No	No	Relatives	120
68	Female	18	Buddhism	No	No	Relatives	110
69	Female	18	Buddhism	Yes	No	Relatives	150
70	Female	19	Muslim	No	No	Two parents	50
71	Female	18	Buddhism	No	No	Two parents	35
72	Female	18	Buddhism	No	No	Two parents	75
73	Female	18	Buddhism	No	No	Two parents	30
74	Female	18	Buddhism	No	No	Two parents	45
75	Female	16	Buddhism	No	No	Relatives	150
76	Female	19	Buddhism	No	No	Two parents	75
77	Female	18	Buddhism	Yes	No	Two parents	120
78	Female	18	Buddhism	No	No	Two parents	45
79	Female	18	Buddhism	No	No	Two parents	45
80	Female	18	Buddhism	No	No	Relatives	15
81	Female	17	Buddhism	No	No	Two parents	150
82	Female	18	Buddhism	No	No	Two parents	60
83	Female	18	Buddhism	No	No	Relatives	50
84	Female	17	Buddhism	No	No	Two parents	75
85	Female	18	Muslim	No	No	Two parents	40
86	Female	17	Buddhism	No	No	Two parents	40
87	Female	18	Buddhism	Yes	No	Two parents	180
88	Female	18	Buddhism	No	No	Relatives	60
89	Female	18	Buddhism	No	No	Two parents	100
90	Female	17	Buddhism	No	No	Two parents	45
91	Female	17	Buddhism	No	No	Single parents	100

**Table 2:** The results of the analysis: theme, sub-themes, codes.

Theme	Sub-theme	Codes
Desire	Releasing sexual desire and stress	-happiness -fulfilling sexual desire -biological need and desire -releasing stress
Relationships	A way to connect with one another	-man and woman uniting to become one -feeling of being connected with partner
	A way to show love	-showing love for each other
Roles	Conditional acts: After marriage	-rights that is granted by marriage -something that is permitted after marriage
	Purposive acts: Reproductive means	-to have children -need of child
	Dutiful acts: Wifely responsibilities	-way to serve man as per his desire
Values	Wife vs. sex worker: The value of virginity	Differences in sex with a wife and with sex workers: a wife has a pure body and provides more pleasure, while sex workers lack virginity and offer less pleasure
	Girlfriend or wife: The burden of future engagement	-Difference in sex with a girlfriend and a wife: with a girlfriend, there's uncertainty about whether we will marry or not."

### 3.1 Desire

#### 3.1.1 Releasing sexual desire and stress

Many participants, including females, agreed that they found sex enjoyable. One male student mentioned that humans, similar to animals, naturally desire fulfilling their sexual needs. Sex is also regarded as a form of stress relief or evening entertainment.

*“And maybe it is our desire. It is normal for men and women to desire sex.” (Soren, female, aged 18)*

*“For general people, some just want to have fun for a while, and they need to fulfill their desire.” (Nita, female, aged 19)*

*“The purpose of sex is to fulfill our sexual desires, and humans have similar feelings as animals. We want to have fun, so we do it to satisfy our desires.” (Kosal, male, aged 19)*

### 3.2 Relationships

Male and female participants expressed that sex was a way to strengthen relationships and that the act of sex became a medium for expressing love toward one another. This theme was used to examine the romantic responses of students to sexual encounters.

#### 3.2.1 A way to connect with one another

One female student believes that sex is a ritual in which two people accepted each other as life partners. She noted that sex serves as a physical promise to their partners, signifying their romantic bond that would endure for the rest of their lives. One male student provided a similar answer, stating that sex evokes feelings of love and happiness. He added that he felt at ease after engaging in sexual activity with his significant other.

*“The meaning of sex is the accepting of a person into my life and us becoming one.” (Chanthavy, female, aged 18)*

*“We need sex to feel happy, in love, and connected with a partner, and able to sleep.” (Khemera, male, aged 18)*

#### 3.2.2 A way to show love

Many female students claimed that they express their affection through sex. To them, the act represents love, trust, honesty, faith, and the commitment to never betray their partners. Furthermore, many were willing to have sex with someone they genuinely loved.

*“I am aware that sometimes it is the display of love. If we are just friends, I will not allow touching or having intimate relationships. However, for the person I love, I will allow it. I will be willing to sacrifice my body to fulfill his desires. This is the meaning of the love that I have shown him. Love is shown through sexual intercourse.” (Bopha, female, aged 18)*

*“I want to show him my faithfulness and sincerity that I will never betray him.” (Chenda, female, aged 17)*

### 3.3 Roles

#### 3.3.1 Conditional acts: after marriage

As provisions of sexual intercourse have been frequently discussed, this theme focuses specifically on marital sex. Some female participants believe that marriage authorizes them to give their husbands something they had previously protected and treasured. Despite the fact that modern norms indicate an increase in the prevalence of premarital sex, many still regard sex as an act of love between husband and wife, such that they should not hesitate to give themselves in such a physical manner in a marital context.

*“It’s meaningful when both of them decide to get married and they can wait till the last night.” (Kiry, male, aged 18)*

*“[Sex is] to show the love and at that time we should not be afraid of anything because we are already husband and wife. So, what I have kept I will give to my husband.” (Deavy, female, aged 16)*

#### 3.3.2 Purposive acts: reproductive means

Many participants mentioned children on this theme. They believe that the purpose of marriage is to have children, which requires sexual intercourse. For these participants, children are the result of their love and an investment in their old age. Some participants claimed that children unite a couple and serve as a means to continue their lineage. One participant felt that he needed someone to care for him as he aged. He regarded the child as a resource to support him during old age.

*“The meaning of sex… is the desire of a man who wants to be with a woman and to have a baby.” (Kunthea, female, aged 18)*

*“I think after marriage we have sex because we want to have children to protect our lineage and receive the result of our love.” (Chantou, female, aged 18)*

*“I think, [the purpose of sex is] to have kids for the next generation. When I get older, they can take care of me.” (Mony, female, aged 18)*

#### 3.3.3 Dutiful acts: wifely responsibilities

Some female students felt that sex would be their duty, as husbands took their wives for that purpose. Some women claimed that marriage was the act of giving life to their husbands. These participants believe that they are willing to sacrifice everything for their husbands after marriage because these men would provide for them for the rest of their lives. As part of this sacrifice, their husbands' sexual desires must be fulfilled.

*“Because it is his desire, I will fulfill it, as we mentioned earlier, whatever he likes and we serve him, something like that.” (Bopha, female, aged 18)*

*“The wife should fulfill her duty as a wife because the husband gets a wife to fulfill his needs.” (Kanya, female, aged 18)*

Additional questions were posed to the female participants. They were asked what they would do if their husbands asked for sex several times a day. While many female participants regarded sex as their duty, they generally responded negatively to the prospect of excessive demand for sex. They felt that excessive sexual intercourse would lead health problems in their reproductive system. Others thought that this would hinder their ability to complete daytime tasks. Many female respondents claimed that unlike previous generations, most modern women participated in economic activities. These obligations, in conjunction with familial duties, meant that they cannot comply with the frequent demands for sex.

*“It is because I cannot have sex with him more than once a day. Sometimes, it exhausts me, and my health deteriorates. My health is more important, so I have to think of it.” (Maly, female, aged 18)*

*“The woman in this present day is not like before, [and] most women now also work outside to earn money. In the olden times she was just a housewife, and had to stay at home, but now it is different.” (Neary, female, aged 19)*

### 3.4 Values

This theme explains the different perceptions of sexual relationships, as they pertain to sex with a wife, sex worker, or girlfriend. A few male students were pleased with the prospect of sexual intimacy with pure and virgin wives. However, they felt psychologically burdened by engaging in premarital sex with a girlfriend due to unclear responsibilities and uncertain future. Furthermore, they were not sufficiently satisfied when engaging in sexual activities with workers.

#### 3.4.1 Wife vs. sex worker: the value of virginity

Several male students clearly distinguished between potential wives and sex workers. However, they focused on virginity as the key factor. According to them, being a virgin is synonymous with having a clean body, which makes sex more pleasurable. These male participants claimed that while their wives would be pure, sex workers would not. Thus, sexual experiences with sex workers are less gratifying. One participant said:

*“Having sex with the sex worker is not romantic because all of them have already lost their virginity and have less sexual pleasure, which is unlike wives or girlfriends who have a clean body because they allow sex only with the person they love. I can say it is more romantic.” (Dara, male, aged 19)*

A few female students mentioned virginity. They believe that they had to offer pure bodies to their future husbands as a bond to unite them.

*“I think he believes in me, [that] I have never done bad things before. And my virginity will be given to him so that he knows his baby is really his and understands that the baby that comes from his blood that belongs to him.” (Sothy, female, aged 18)*

#### 3.4.2 Girlfriend or wife: the burden of future engagement

While discussing their girlfriends, a few of the male students focused on the possibility of marriage. They expressed anxiety about having premarital sex due to the uncertainty of the relationship in the future. These male participants explained that engaging in sexual activities with a girlfriend may lead to unexpected pregnancies or STIs. Furthermore, they described it as being out of wedlock; thus, less meaningful to them.

*“Having sex with a girlfriend, we are not sure if we can be responsible or marry her. Unlike having sex with a wife, which is to have children and create a family together.” (Manndy, male, aged 19)*

## 4 Discussion

This study investigated the social and cultural perspectives on sex among adolescents in Cambodia. The influence of Cambodian culture on adolescents' sexual concepts and behaviors is a key outcome of this research. This study demonstrated differences and similarities in attitudes and perspectives of young men and women regarding sex.

Our study found males and females agreed that sexual intercourse grants them happiness and satisfaction, whereas for women, it is a marital obligation. Most of the respondents who focused on sex for physical pleasure were male. Men often focus on the physical aspects of sex, whereas women focus on the emotional aspects. Some male students acknowledged the emotional exchanges involved in sexual intercourse. These participants agreed that sex strengthened their relationship with their partners, allowing them to express their love for one another and feel connected.

Previous study reported that sex was considered a women's obligation (21). Our study result also revealed some female adolescents expressed that wives were obligated to have sex with their husbands. Others felt a strong sense of duty toward their role as wives, particularly concerning sexual matters. These beliefs are driven by societal and cultural norms that encourage acquiescence toward a husband's sexual desires (23).Some male participants ascribed different values to sex, depending on their sexual partner (wife, girlfriend, or sex worker). In Cambodian culture, a girl's values vary on the basis of her purity (24), and women are taught that a wife should retain her vaginal purity until marriage. Our study found many believed that a “pure” woman is dedicated entirely to her husband, which increases sexual gratification. Male adolescents expressed that they would feel more comfortable having sex with a future wife than with their girlfriends. Among the various sexual services, paid services for sex workers were the only ones available to experience sexual release. Interestingly, not all women view sex workers negatively. Previous literature reported that some wives believe they are incapable of sexually pleasing their husbands; thus, they encourage their husbands to have sexual relations with other women to avoid divorce (23). Additionally, men often perceive sex as innate and vital for personal happiness. In Cambodia, engaging with sex workers is a prevalent cultural norm, and Cambodian society does not view it negatively (19). However, sex workers, of which there are an estimated 40,000 nationwide, are considered a high-risk group for STIs (25). Recently, the prevalence of STIs in Cambodia has significantly decreased as a result of the government's national efforts to protect sex workers from STIs (26). However, despite those efforts, men often spread STIs to their wives when they do not use condoms during sexual intercourse (19).

To prevent STI transmission in Cambodia, the perception of sexual labor must be altered. The fulfillment of masculine needs is often used to justify engaging with sex workers; however, these men must be made aware of the dangers of STIs. Furthermore, those at risk of spreading STIs should be educated on the essential use of condoms during intercourse, even with their wives, to prevent transmission. Thus, rather than demonizing sex workers, STI cross-infection must be minimized.

Cambodia has a unique culture that includes sexual behavior among adolescents and young adults. Although Cambodian society has changed under the influence of Western culture, this study revealed that some Cambodian adolescents retain traditional attitudes toward sexual behavior. Furthermore, Cambodian men have more sexual and general freedom, whereas women are expected to retain their “purity” for their future husbands. Additionally, male sex before marriage is widely accepted by male and female adolescents.

The level of sexual concept varies depending on the meaning and purpose of sex, and in some cases, the risk of exposure to early sexual experience, lack of condom use, and maintenance of multiple sex partners may increase. Indiscriminate sex, caused by low-level sexual concepts, can be a shortcut to facing uncomfortable situations (e.g., STIs, unexpected pregnancies, and so on.) Adolescents and young adults should be taught that seeking sexual health care is necessary and normal to protect their health and that of their loved ones. Systems should also be established wherein adolescents can access regular check-ups. As in many developing countries, access to health care in Cambodia is constrained by poverty. By understanding Cambodian youth's perspective on sex, we can better understand the prevalence of STIs and the causes of unexpected pregnancies among adolescents in Cambodia. Several educational programs and resources are available to inform Cambodian citizens about safe sex practices and encourage the use of health services. However, STIs continue to spread from husbands to wives. Therefore, revised and culturally accepted sex education and intervention programs must be implemented to prevent the spread of STIs. Ultimately, adolescents in Cambodia must be taught appropriate SRH education.

## 5 Strengths and limitation of the study

A notable strength of this study is its adherence to rigorous qualitative research methodology, conducted across three regions capturing the perspectives of approximately 100 high school seniors. They eloquently expressed their thoughts and viewpoints, allowing for a comprehensive understanding of perspectives within Cambodia's unique culture and societal context. This can be considered a pioneering study in this regard. However, this study interviewed adolescents in rural areas only. Therefore, future study should include adolescents in urban areas to capture any changes in perception on sex among Cambodian adolescents nowadays. While language limitations may arise due to the principal investigator being a non-Cambodian researcher, over five years of residence in Cambodia and extensive research conducted within the country contribute to overcoming this challenge, supported by knowledgeable and skillful local scholars.

## 6 Conclusion

This study was conducted to comprehend the perspectives on sexuality among Cambodian adolescents. The research findings revealed differences between the viewpoints of males and females, but they were not significantly different from those of adults. To prevent HIV, STIs, and teenage pregnancy, there is a need for comprehensive sexual education promoting healthy attitudes towards sexuality and practicing protected sex. As the internet and social media continue to advance, contemporary adolescents exposed to these mediums require ICT-based educational resources and content. To support this, various research initiatives and policies should be in place.

## Data Availability

The original contributions presented in the study are included in the article/Supplementary Material, further inquiries can be directed to the corresponding author.
